# Expression of E-Cadherin in Pig-Tailed Monkey (*Macaca nemestrina*) Endometrium after Controlled Ovarian Hyperstimulation

**DOI:** 10.1155/2021/8824614

**Published:** 2021-02-23

**Authors:** Nurhuda Sahar, R. Muharam, Andhea Debby Pradhita, Rosalina Thuffi, Wa Ode Zulhulaifah, Ponco Birowo

**Affiliations:** ^1^Department of Biology, Faculty of Medicine, Universitas Indonesia, Jakarta, Indonesia; ^2^Department of Obstetrics and Gynecology, Faculty of Medicine Universitas Indonesia, Dr. Cipto Mangunkusumo General Hospital Indonesia, Jakarta, Indonesia; ^3^Master Program Biomedical Sciences, Faculty of Medicine, Universitas Indonesia, Jakarta, Indonesia; ^4^Department of Urology, Faculty of Medicine Universitas Indonesia, Dr. Cipto Mangunkusumo General Hospital Indonesia, Jakarta, Indonesia

## Abstract

An increase of steroid hormones in controlled ovarian hyperstimulation (COH) procedures is reducing the success rate in assisted reproductive technology (ART), and this includes the pregnancy rate and/or implantation rate. Research has found that the decrease in the success rate occurred due to the decreased expression of the protein that is needed to prepare the endometrium so that the embryo could attach. The aim of the study was to analyse the changes in E-chaderin expression due to COH and its relations with increased level of steroid hormones as one of the proteins in the endometrium. There were 13 samples of stored biological tissue from *Macaca nemestrina* endometrial tissue; came from one group of natural cycles as the control group (*n* = 4) and three groups of stimulated cycles. The first stimulated cycle group was injected by a 30 IU dose of rFSH (*n* = 2). The second stimulated cycle group was injected by a 50 IU dose of rFSH (*n* = 4). The third stimulated cycle group was injected by a 70 IU dose of rFSH (*n* = 3). The expression of E-cadherin was measured by the immunohistochemistry (IHC) technique. Estradiol (E2) and progesterone (P4) levels were assessed using ELISA and have already been done. The IHC staining expression of E-cadherin was found in the cytoplasm of glandular epithelium. Immunostaining measurement used the H_SCORE. We found that the expression of E-cadherin within the group was not significantly different (*p* value: 0.178). Similarly, both the correlation between the estradiol level with E-cadherin and the correlation between the progesterone level with E-cadherin were not significantly different (*p* value: 0.872 and *p* value: 0.836). The conclusion is that the level of E-Cadherin expression in the endometrium that were taken in themiddle secretion phase not affected by the dose regimen that given. In addition, the level of expression is not influenced by the increase of serum E2 and P4 levels.

## 1. Introduction

An increase in steroid hormones in the late follicular phase is common in COH procedures [1, 2]. That condition usually described as an indicator of the COH procedure on the development and maturation of the ovarian follicles [3]. The production of a large number of mature follicles is one of the factors that play a role in increasing the success of IVF [4]. Unfortunately, high levels of steroid hormones have a negative impact on the endometrial receptivity of the implantation window [5]. The low success percentage of implantation and pregnancy in the IVF program is likely due to a hormonal imbalance in the blood [6].

Endometrial receptivity is a limited period of time in the secretory phase of the menstrual cycle which the endometrium can accept the presence of an embryo [7]. Within 28 days of the menstrual cycle, the period of acceptance for endometrial implantation occurs in the middle luteal phase, which is the 20th to the 22nd day [8]. The endometrium changes morphologically or molecularly during the implantation period or implantation window [9]. The morphological change is the cytoplasmic elongation of luminal epithelial cells towards the lumen known as pinopod [10]. Biochemically, the receptive period of the endometrium is characterized by maximal expression of several adhesion protein molecules found in the endometrial luminal epithelium [11]. Among the proteins expressed in the endometrial luminal epithelium are *β*-catenin, CD166/ALCAM, glycodelin A (GdA), leukaemia inhibiting factor (LIF), stem cell factor (SCF) and its receptor (c-Kit), epithelial growth factor (EGF), Mucin-1 (MUC-1), integrin *α*V*β*3, and insulin like growth factor (IGF) [12–15].

Epithelial cadherin (E-cadherin) is a glycoprotein transmembrane, which is a member of the adhesion molecule family. E-cadherin serves as a bond mediator between the embryo and the endometrial epithelium [16]. E-cadherin is detected by an immunohistochemical technique in the cytoplasm of glandular and luminal epithelia. The expression begins to increase after ovulation and reaches the optimum as long as the implantation period [17]. E-cadherin is a marker of endometrial receptivity that binds the embryo to the endometrial epithelium [18]. Ovarian stimulation decreases E-cadherin expression [19]. Steroid hormones that increase the implantation period after COH had been given [20]. Estradiol has been reported to reduce the regulation of E-cadherin in several reproductive tissues, including the uterus [21]. The disrupted expression of E-cadherin by estradiol causes pregnancy failure [22]. In this study, we have evaluated the expression of E-cadherin in the midluteal phase of *Macaca nemestrina* endometrium after controlled ovarian hyperstimulation. The aim is to determine the effect of various doses of an ovarian stimulator on the expression of E-cadherin and its correlation with increased estrogen and progesterone secretion.

## 2. Methods

### 2.1. Animals

Animals used in this retrospective experimental study were females (*Macaca nemestrina*) at reproductive age (8-10 years) that have an average body weight 5-8 kg and have already given birth. The animals were obtained from the Primate Animal Study Center, Bogor Agricultural University, Bogor, Indonesia. The study protocol was approved by the Institutional Animal Care and Use Committee for Primate Animal Studies, Bogor Agricultural Institute.

The animals chosen for use in this study were tattooed with identification numbers in the growing area and placed in individual cages made of stainless material. All animals were quarantined and adapted to new individual cages for two to three menstrual periods. Animal health is maintained and any care is given as needed.

We used 13 animals divided into four groups. The number of animals approved has been determined by the ethics committee. The first group was as a control group (*n* = 4) where the animals were not got injection of recombinant-FSH (rFSH), and three others groups were stimulated group where the animals were injected by rFSH. The stimulated groups used controlled ovarian hyperstimulation (COH) procedures with 3 different doses of rFSH and the same dose of GnRH and hCG. The first stimulated cycle group was injected with a 30 IU dose of rFSH (*n* = 2), the second stimulated cycle group was injected with a 50 IU dose of rFSH (*n* = 4), and the third stimulated cycle group was injected with a 70 IU dose of rFSH (*n* = 3).

### 2.2. Controlled Ovarian Hyperstimulation Procedure (COH)

**Figure 1:**
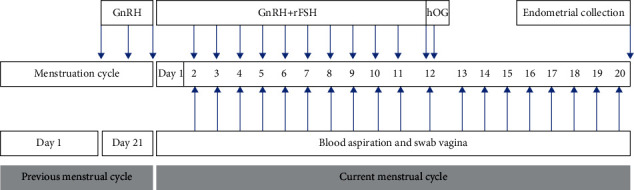
Controlled ovarium hyperstimulation (COH) procedure.

For the COH procedure, a combination of gonadotrophin was given with the long GnRH protocol using one of the following three regimens, that were (1) recombinant follicle stimulating hormone (rFSH) (Gonal F; Merck KGaA, Darmstadt, Germany), (2) GnRH agonist (Suprefact; Sanofi S.A., Paris, France), and (3) human chorionic gonadotropin (hCG) (Pregnyl; Merck KGaA) (Figure 1).

The GnRH agonist administered at a dose of 160 *μ*g/day began in the middle of luteal phase in previous menstrual cycle and continues until the day before ovulation (about 14 days given). After E2 hormone levels are less than 70 pg/mL on the second day of menstruation, we combined the therapy with rFSH in each stimulated group. The first group received a dose of 30 IU, the second group received a dose of 50 IU, and the third group received a dose of 70 IU of rFSH. The rFSH was injected on the second day after menstruation at doses according to the treatment group for 10 days until the peak secretion of the E2 hormone occured. Furthermore, we administered 10000 IU of hCG, equivalent to 3200 IU. The luteal phase was determined by measuring serial P4 levels that begin on the postovulatory day.

### 2.3. Blood Sampling

Blood samples were collected from the second day after menstruation until the middle secretion phase (21st day of the menstrual cycle) ait intervals of every two days. As total of 5 mL of blood is collected from the femoral vein in the groin area. Blood was centrifuged at 2500 rpm for 15 minutes. Serum was separated into new polypropylene tubes and stored at -20°C before measurement.

### 2.4. Hormone Assay

We used the data from the measurements of the E2 and P4 hormones that we had previously worked on. We used serum for the testing of E2 which was taken on the day of hCG injection assumed that E2 reached its peak level at the same day as the day of hCG injection. We used serum for pP4 testing on the same day as the uterine tissue collection.

E2 and P4 were measured by the Chemiluminescent Competitive Immunoassay (IMMULITE, DPC, Los Anges, CA, USA). The sensitivity of the E2 assay was 10 pg/ml and the intra-assay cofficient of variation was 5%. The sensitivity of the P4 assay was 0.2 ng/ml and the intra-assay with cefficient of variation was 6.7%. The colour that arised due to hydrolysis of the alkaline phosphatase enzyme on the substrate was measured with a luminometer. The polyclonal steroid antibody bound to the beads was incubated together with a second antibody coated with an alkaline phosphatase enzyme and serum samples for 60 minutes at 37°C. Blood was allowed to clot, and serum was separated and stored at -20 °C until assayed.

### 2.5. Endometrial Collection

The uterus of each animal was collected 9-10 days after the peak of estradiol secretion. Before surgery, each animal was anaesthetized with ketamine at a dose of 0.1 mL/kg body weight. In necropsy, the whole cut of the uterus was rinsed with phosphate buffer, incubated in 10% formalin solution, and implanted in paraffin blocks.

### 2.6. Hematoxylin-Eosin Staining

The hematoxylin-eosin (HE) staining procedure is to deparaffinize using xylol, rehydrate using alcohol, and wash in running water. Preparations were put into Mayer's hematoxylin solution, rinsed with running water, and dipped into saturated lithium carbonate. Slides were rinsed with running water for 5 minutes. The staining was used to make sure that the right tissue was identified.

### 2.7. Immunohistochemistry for E-Cadherin

The endometrial tissue embedded in paraffin blocks were cut into thin slices with a thickness of 0.3-0.5 *μ*m. They were affixed to the glass object that has been coated with APEX. The paraffinization process was carried out in xylol solution and continued with dehydration in an alcoholic solution. Slides were washed in a 0.05 M PBS solution pH 7.2. Slides were incubated for 10-15 minutes in H_2_O_2_ solution and washed with water. Slides were placed in the retrieval buffer solution and were heated for 30 minutes in Retrieval Generation One (RG1) for 30 minutes. After cooling, slides were washed with running water and Rabbit anti E-chaderin polyclonal antibody (Bioenzy) was added at a dilution of 1 : 100. Slides were incubated for 60 minutes at room temperature. Slides were washed with buffer in solution. Polymer HRP was added to the slides, which were then incubated for 25 minutes at room temperature and washed again in PBS solution. We added one drop of dye and left it for 5 minutes. Slides were washed with running water. Slides were incubated for 1 minute in HE staining and were washed with running water. We closed the slide with mounting media and observed them under a light microscope.

We used Ca mammae tissue as a positive control and normal endometrial tissue as a negative control to compare the result of the samples. Slides were seen under light with 400x magnification. We selected five areas randomly and photographed them using a camera. We calculated all the numbers of cells and determined the intensity of the brown colour used ImageJ analysis software.

We used the formula H_SCORE = *Σ*pi (*i* + 1) as a semiquantitative score; pi is the percentage of coloured cells (value: 0-100%); and *i* is the colour intensity. Strong intensity was given a positive score of +3, moderate intensity a score of +2, weak intensity a score of +1, and a score of 0 if the coloure were not occured. The score from each slide was the average number of the positive coloured score and colour intensity.

### 2.8. Statistical Analysis

The data were described in the form of median and maximum-minimum values. Comparison of immunoreactive scores between groups given GnRH stimulation and rFSH 30 IU, 50 IU, and 70 IU, and the natural control group were analysed for the significance of the difference using the SPSS 22 software. The first step in this analysis was the normality and homogeneity of the data. If the distribution was normal and homogeneous, the statistical test used was one-way ANOVA. If the analysis with ANOVA showed significant differences, the analysis continued with the Tukey HSD (Honestly Significant Difference) test to find out the differences between one and another group. If the data distribution was not normal and not homogeneous, the test was conducted by the Kruskal-Wallis test.

If the data was normally distributed, we used correlation analysis with the Pearson correlation. If the data was not normally distributed, we used correlation analysis with the Spearman correlation. The statistical test decision uses a 5% significance level (*p* = 0.05).

## 3. Result

### 3.1. Results of E2, P4, and E-Cadherin

Data are presented with median (minimum-maximum) values because our data are not normally distributed and the numbers are too small (Table 1).

We used serum E2 data taken at the late follicular phase. We assessed an increase in all three stimulated groups compared to the control group. The P4 serum we took was at around the midluteal phase. From the result, only the 50 IU stimulated group had a higher median value than the control group. The 30 IU and 70 IU stimulated groups had lower median values than the control group. The E-cadherin values were taken over the network during the midluteal phase and given IHC staining. The median E-cadherin value obtained showed that the expression in stimulated group were lower than the control group.

### 3.2. Hematoxylin-Eosin Staining

**Figure 2:**
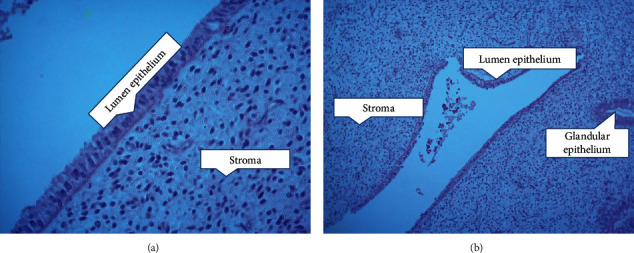
Endometrial histology image on days 8-9 after peak estradiol levels with hematoxylin-eosin staining. (a) 400x magnification. (b) 100x magnification.

We made sure this cut tissue was the correct tissue for immunohistochemical staining (Figure 2). We assessed the presence of glandular mitosis, stromal edema, stromal mitosis, vacuoles, and leukocyte infiltration. Menstruation on days 20-24 has increased stromal proliferation and enlarged spiral arteries [23].

### 3.3. Results of IHC Staining on E-Cadherin

**Figure 3:**
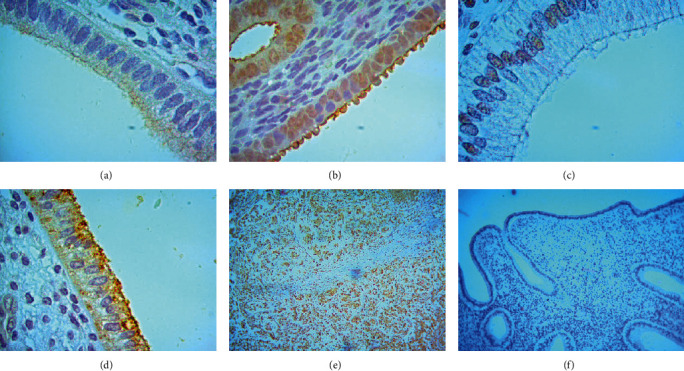
E-cadherin expression on IHC staining. Positive staining showed in the cytoplasm of the glandular epithelium of the endometrium samples. (a) The stimulated group with 30 IU rFSH; (b) The stimulated group with 50 IU rFSH; (c) The stimulated group with 70 IU rFSH; (d) Control group; (e)Positive control of ca mammae; (f) Negative control.

**Figure 4:**
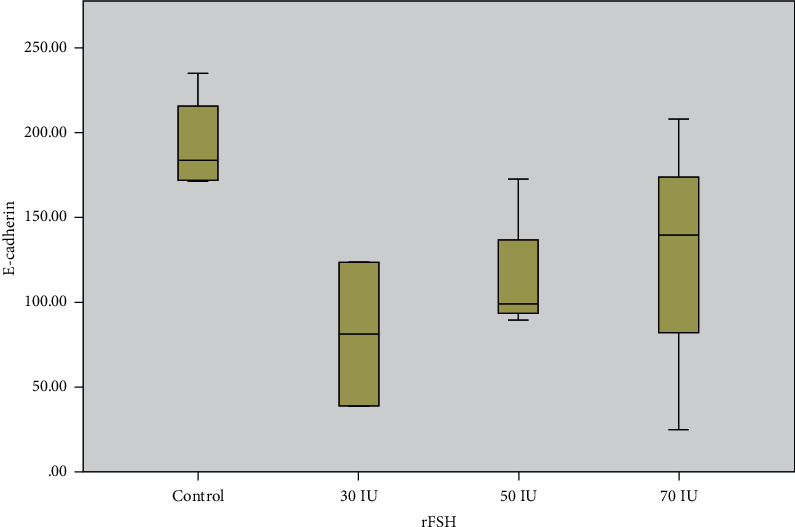
Comparison of E-cadherin expression in the endometrial control group and the stimulated groups. There were no significant differences between the four groups. Analysis using the Kruskal-Wallis test (*p* > 0.178).

E-cadherin cellular distribution and quantification were assessed by immunohistochemical staining. The location of expression is detected in the cytoplasm of the luminal gland epithelial cells. A total of 13 samples were examined, and all tested positive with the intensity of the brown colour that appeared on the immunohistochemical staining. The weak intensity that appears varies from weak to very strong intensity.  Figure 3 shows the level of E-cadherin expression in the endometrium of *Macaca nemestrina* in the natural and stimulated groups. The intensity of the brown colour that appeared was stronger in the natural group than in the stimulated group. Using the H_SCORE formula (Figure 4), the mean level of E-cadherin expression was high in the control group, then low in the 25 IU dose treatment group but high at the 50 and 70 IU dose treatment groups (Table 1).

### 3.4. Comparison of E-Cadherin Expression between the Control Group and the Stimulated Groups

From the boxplot presentation (Figure 4), the median of the control group was higher than those of the three stimulated groups. The minimum and maximum values of the three stimulated groups were lower than that of the control group (*p* value: 0.178).

### 3.5. Correlation Analysis between Late Follicular Phase Estradiol and Midluteal E-Cadherin

The correlation of E2 with E-cadherin was very low in a negative direction although there were no significant differences (Table 2). The coefficient correlation of E2 and E-cadherin was -0.05 with a *p* value of 0.872.

### 3.6. Correlation Analysis between Midluteal Progesterone and E-Cadherin

The correlation of P4 with E-cadherin was very low in a positive direction although there were no significant differences (Table 3). The coefficient correlation of P4 and E-cadherin was 0.064 with a *p* value of 0.836.

## 4. Discussion

The success of IVF is determined based on proper communication between the embryo and the endometrium [24]. Proper communication cannot take place throughout the menstrual cycle. Successful IVF requires preparation of the follicles for ovulation, proper hormone levels, a good quality embryo, and endometrial receptivity. All of these lead to the time of acceptance of the embryo which is the implantation window. The implantation window has a limited time for embryo acceptance and is accompanied by support from the endometrium (hormones, maternal immunity, and other factors) [24]. The steroid hormone, such as E2, P4, and hCG, support the endometrium during the implantation window. Estradiol functions during the follicular phase or the proliferative phase during follicular development. Progesterone increases during the luteal phase or secretion phase to support a comfortable endometrium for the embryo [24].

### 4.1. Factors Affecting the Implantation Window

Many processes are not fully understood when the implantation window occurs. The implantation window determines the molecules that affect the signal pathway for endometrial maturation. At that time, stromal cells differentiate into decidual cells first modulated by progesterone, and then other cell types and molecules interact to allow the coordination of embryo apposition, attachment, and invasion of the endometrium [9, 25, 26].

Preparation for implantation begins when the menstrual cycle begins. The proliferative phase at the beginning of menstruation increases E2 levels as a result of developing follicles. The E2 produced will attach to E2 receptors in the endometrium for stromal proliferation, epithelium proliferation, and vascularization as functional regeneration of the post-menstrual endometrium. After ovulation, the corpus luteum produces P4 which prepares the endometrium for maturity at the implantation window. PE will induce pinopod development. A mature pinopods will reduce the amount of microvilli on their surface and reduce the amount of Mucin-1 (MUC-1) [27].

Implantation begins with apposition, where the hatched blastocyst will go to a location in the endometrial lumen. The next stage is adhesion, namely, the presence of cellular communication from the surface of the endometrium with blastocytes. After that, there is a deep invasion through the endometrium. Prostaglandin E2 is increased as a local inflammatory response to support inflammation. There is a change in the stroma to support embryo attachment which is called decidualization. Cytokines will increase for cell communication. Leukaemia Inhibitory Factor (LIF) is one of the important cytokines in regulating decidualization. LIF regulates the epidermal growth factor (EGF) signaling pathway. Colony-stimulating factor-1 (CSF-1) is a gene that increases embryo-endometrial attachment. Interleukin-1 (IL-1) is a cytokine that stimulates vascular endothelial growth factor (VEGF) expression and regulates matrix metalloproteinases (MMPs) and tissue inhibitors of metalloproteinases (TIMPs). Interleukin-6 (IL-6) functions on the endometrial epithelium. Heparin-binding epidermal growth factor-like growth factor (HB-EGF) also functions for embryo-endometrial interactions [27]. Cell adhesion molecules such as integrins, E-cadherins, and L-selectin play a very important role during apposition and adhesion [28]. Cell adhesion molecules are expressed on the surface of the invasive trophoblast and interact with ligands expressed by the extracellular matrix of the decidua. During the invasion, the trophoblast embryo will enter deeper into the endometrium to a part of the myometrium. Invasion is for reconstructing the maternal spiral arteries, which will maintain a high blood flow between the fetus and the mother, replacing small, high-resistance vessels with large, low-resistance vessels. Then, it will form placental villi assisted by remodeling of the extracellular matrix, namely, matrix metalloproteinases (MMPs) and collagenases [27].

### 4.2. E-Cadherin as a Marker of Endometrial Receptivity

E-cadherin is a cadherin epithelium, one of the classic cadherins, which is a glycoprotein that is on the surface of cells responsible for implantation and changes in the structure of the embryo. Cadherin is one of adhesion molecules expressed in glandular and luminal epithelia. Through a calcium-dependent mechanism, E-cadherin maintains bonds between cells [29].

Cadherin interacts with catenin which will connect cadherin with the actin cytoskeleton. The interaction of *α*-catenin with the actin cytoskeleton is also important for regulation. *α*-Catenin interacts with a number of actin-binding proteins, including *α*-actinin, vinculin, and ZO-1. Tyrosine phosphorylation of the cadherin-catenin complex is also involved in the regulation of adhesion originating from kinase activation. The p120ctn protein, which is structurally related to *β*-catenin (a protein that contains repetitive armadillos), is also a regulator of adhesion activity. It binds to an area of the cadherin cytoplasmic tail, the juxtamembrane domain, which differs from the classical catenin binding site. Small GTPase, Rac, Rho, and Cdc42 are also involved in cadherin-mediated adhesion. [30]

Another potential factor related to E-cadherin expression is HOXA 10. HOXA 10 is known to be linearly related to E-cadherin through studies by Yang et al. HOXA 10 belongs to the HOX genes, namely, the Homeobox gene which is a transcription factor. HOXA 10 is a response to the E2 and P4 hormones during the midluteal phase. Then, HOXA 10 will regulate E-cadherin expression in the endometrium [18]. Due to increased P4 at the midluteal phase, endometrial calcitonin can induce an increase in intracellular calcium which can increase E-cadherin expression [8, 31].

### 4.3. Steroid Hormones between Normal Menstrual Cycle and Stimulation Groups

Is one of the most abundant and most active estrogen hormones during the menstrual cycle. We measured peak estradiol levels at the same time as the day of hCG injection. We estimated that ovulation occurs 18 hours after hCG injection. It shows that the three groups who underwent COH had higher E2 levels than the control group who had normal menstruation (Table 1).

The measured P4 is the level of progesterone that is taken when the uterus is extracted. We estimate that 8-9 days after the peak of E2 levels are due to the midluteal phase. The midluteal phase is the time for the implantation window, which is around days 20-22. However P4 in the four groups has varied results (Table 1). There is no conclusive pattern in the control group or the stimulated group. The rFSH stimulation dose group of 30 IU and 70 IU had lower results than the control group, while the 50 IU rFSH stimulation dose group had higher results than the control group. Van der Gaast et al. said that there was no difference in P4 levels during the luteal phase in both the natural cycle and stimulated cycle groups [32].

In the 50 IU rFSH dose group, there was a difference compared to the other 2 stimulation groups. This may be due to exposure to additional hormones that affect P4 production by a corpus luteum not undergoing functional luteolysis, and may have retained some of its ability to produce P4 under stimulation by endogenous and exogenous gonadotropins in the stimulated cycle [33]. The occurrence of variations between low E2 and high P4 can occur in cycles that are given ovarian stimulation [34].

In the administration of rFSH, there was no significant difference at each dose given because every samples have an individual response. This does not mean that a higher dose will produce a better output. In a study conducted by Tian et al., they divided the high dose and the low dose using a long protocol. The results were not significantly different. We drew conclusions that the rFSH dose group produced different E2 and P4 outputs than the other rFSH group [35].

Wen et al. studied about the levels of steroid hormones (E2, P4, and testosterone) after taking oocytes. Their studied showed that steroidogenesis was associated with increased gonadotropins. They assessed follicular fluid and granulosa cell cultures. As a result, they found that ovarian stimulation can cause the occurrence of pre-ovulation luteinization, and levels of E2 correlate with P4 levels [36].

### 4.4. Comparison of E-Cadherin Expression between Normal Menstrual Cycle and Stimulation Groups

Research assessing the endometrial midsecretory phase of infertile and endometriosis patients shows higher expression of E-cadherin protein in endometriosis compared with controls of healthy fertile women. These results suggest that downregulation of E-cadherin during the implantation window is a potential mechanism for implantation that allows epithelial cells to dissociate and invade the blastocyst [37]. E-cadherin is expressed in the cytoplasm/epithelial membrane. Valdez-Morales et al. said that there were no significant differences between groups, one of which used the rFSH regimen [29].

It can be seen that the control group had a higher median than the stimulation group (Figure 4). The higher dose stimulation group had higher E-cadherin expression, although not significantly different. Similar to our research, research by Maia-Filho did not show significant differences between the IVF group and the group with natural menstruation [38].

We used the H_SCORE in the immunohistochemical assessment. There was a difference in colour intensity even though it was very slight, where a darker colour indicates a more positive result and indicates the amount of E-cadherin in the endometrial lumen epithelium. This result was similar to the study conducted by Chakravarty et al., they also found no statistical difference in E-cadherin in the endometrial lumen. They said that the success of IVF should be linear with the amount of E-cadherin because it indirectly shows the timeliness of the embryo transfer with the Window of Implantation (WOI) [39].

### 4.5. Correlations between Steroid Hormones and E-Cadherin

When the endometrium is receptive, the regulation of steroid hormones is important in regulating endometrial morphology. We assessed the relationship between E2 and E-cadherin and P4 with E-cadherin.

In our study, there was no significant correlation between E2, P4, and E-cadherin (*p* > 0.05). This research was in line with research conducted by Kiewisz et al. who used pregnant pigs as experimental animals. In their research, there were also no correlations between E2, P4, and E-cadherin, although the direction is negative [40].

According to the theories that we learned, in the endometrium, the levels of E-cadherin and *β*-catenin mRNA increased after ovulation but than reduced by the E2. E2 reduces cell-to-cell adhesive function, and P4 stimulates it [41]. But that condition was not shown in this research. The other studied said that E2 will activate the receptor and the E2 receptors found to have direct correlation with E-chadheri expression [42]. This might be the reason why the correlation of E2 with E-chaderin could not shown signficantlly different. E2 might be not has direct correlation, but the receptor has.

### 4.6. IVF Regiments and Implantation Windows

Estrogen levels in the stimulated group were higher than the control group because in the stimulated group there was an increase in the number of follicles. The increased estrogen will affect angiogenesis, gene expression, and endometrial receptivity and can influence implantation success [43]. Recovery of the hormones produced by the pituitary gland after administration of GnRH agonists to the ovarian hyperstimulation regimen will occur after 14 days [44]. The shifting of the implantation window due to progesterone that has already increased can affect endometrial receptivity if it is more than 3 days [1]. Simon et al. said that during IVF, lowering estrogen before embryo transfer time will increase success [45].

Progesterone levels in the luteal phase greatly affect the success of implantation. The implantation window occurs at the peak of progesterone around days 6-8 after ovulation. Shifting of the progesterone peak during the midluteal phase affects endometrial receptivity due to the shifting of the implantation window. The endometrial out-phase is a state of the endometrium not in accordance with the conditions that should occur according to the menstrual cycle [46].

Wang et al. said that the endometrial histology taken by biopsy in IVF/ICSI patients had a change in receptivity so that implantation failure and miscarriage often occurred. They assessed the endometrium from volumetric fractions of vascular endothelial cells. Blood vessels that are too small or too large can affect receptivity [47].

We did not assess follicle size via ultrasonography when the injection of the ovarian stimulation regimen was administered, so we cannot confirm whether follicular maturation has occurred and whether it was followed by the occurrence of the luteal phase. Endometrial development is strongly influenced by steroid hormones. Giving ovarian stimulation is thought to affect endometrial displacement and can affect the timing of the implantation window.

## 5. Conclusion

The expression of E-cadherin is not controlled by E2 and/or P4. E2 and P4 do not directly affect E-cadherin activation. We did not find any difference in steroid hormone or E-cadherin levels in the stimulated or control groups. We did not find any correlation between steroid hormones (E2 and P4) and endometrial E-cadherin in the luteal phase as a marker of endometrial receptivity. We need further research to answer this.

## Figures and Tables

**Table 1:** The descriptive result of hormone E2, hormone P4, and E-cadherin adhesion molecules.

	Control group	Stimulated group		
	(*n* = 4)	30 IU (*n* = 2)	50 IU (*n* = 4)	70 IU (*n* = 3)
E2	530.5 (426-706)	931.5 (107-1756)	776 (610-3000)	1319 (772-1913)
P4 midluteal	3.35 (2.4-4.9)	1.94 (0.3-3.5)	4.25 (0.3-9.9)	1 (0.3-5.3)
E-cadherin	183.7 (171.5-235)	81.2 (39-123.5)	99.2 (90-172.5)	139.5 (25-208)

**Table 2:** The results of the correlation analysis between E2 hormone and E-cadherin.

	E-cadherin
E2	*p* = 0.872
	*r* = −0.05
	*n* = 13
Spearman's correlation	

**Table 3:** The results of the correlation analysis between P4 hormone and E-cadherin.

	E-cadherin
P4	*p* = 0.836
	*r* = 0.064
	*n* = 13
Spearman's correlation	

## Data Availability

The data used to support the findings of this study were supplied by Nurhuda Sahar. Requests for access to these data should be made to Nurhuda Sahar, nurhudasahar@yahoo.com.
